# Role of Politics, Guilds and Pedagogy in defining Policies in Medical Education: The Pakistan Scenario

**DOI:** 10.12669/pjms.38.6.6057

**Published:** 2022

**Authors:** Lubna Ansari Baig, Syeda Kauser Ali, Shaur Sarfaraz

**Affiliations:** 1 Prof. Lubna Ansari Baig, MBBS, MMEd, MPH, FCPS, PhD. Founding Dean and Chairperson, APPNA Institute of Public Health Jinnah Sindh Medical University, Karachi, Pakistan; 2 Prof. Syeda Kauser Ali, MBBS, MHPE, PhD. Chairperson, Institute of Medical Education, Jinnah Sindh Medical University, Karachi, Pakistan; 3 Dr. Shaur Sarfaraz, BDS, MBA (HCM), MHPE. Assistant Professor, Institute of Medical Education, Jinnah Sindh Medical University, Karachi, Pakistan

**Keywords:** Pedagogy, Politics, Guilds, Market forces Medical Education

## Abstract

Politics in education is not a new concept and has been a sore point of discussion between academia and policy makers. The politics of medical education has led to the formation of informal guilds that have taken control over medical education. Policy decisions concerning medical/dental education affecting the lives of the community, at large are implemented without giving due consideration to the pedagogy of medical education. This paper delves into the definitions of politics, pedagogy and guilds and with evidence identifies that major policy decisions in medical education are strongly influenced by politics. The paper will discuss that politics of medical education is not necessarily a bad thing if it ensures public safety and is based on best evidence medical education. In the same context the guilds formed for medical education reforms should uphold the principles of pedagogy.

## INTRODUCTION

The role of politics’ has been witnessed internationally in professional education with an ongoing struggle in decisions for student admission, the choice of curriculum, teacher appointment and promotions, licensure etc.^1^ One needs to understand who makes these decisions, how they are made and how these decisions impact the lives and professional growth of the stakeholders, especially the students, their families and teachers.

The health professionals in Pakistan are facing difficulties in registration with licensing bodies and training abroad with the frequent and abrupt changes happening at the level of Pakistan Medical Commission (PMC), which was previously Pakistan Medical and Dental Council (PMDC).^2^ There has been pressure from accrediting bodies to change from a discipline-based to an integrated, community-oriented curriculum. Concurrently the system of education was changed from annual to semester-based and then reverted to the annual system. There is no evidence to show that these changes were based on any scientific rationale or need assessment in the national context. The common perception is that they were driven by mere political pressures and /or market forces.

Although politics and market forces are inevitable realities and cannot be ignored, there is a need to align them with community needs and public expectations and create a win-win situation for all stakeholders. The power of politics should be used positively rather than using it as a negative notion. The Medical Professionalism (MP) project (2002), states that participation of political forces for medical advocacy ensuring better community health through collective efforts are sometimes needed. The authors of the MP project concluded that the commitment to professionalism should not be surpassed by politics, and pressures from market forces.^3^ Huddle TS however in his perspective on professionalism argued that politics and advocacy should not be the role of the medical profession. He further argued that politics may not be innocent all the time and universities should refrain from such diversity.^4^ However, we believe that the market forces and an appropriate use of politics in line with educational pedagogy can be used for medical reforms for ensuring better health of the community.

### History of Medical Education in Pakistan

In Pakistan, the Traditional Curriculum set by British was followed by almost all medical colleges till late 90’s until the introduction of integrated curriculum, Community-Oriented Medical Education (COME) and Problem-based Learning (PBL). A number of public and private sector institutions in Pakistan initiated curricular revisions and introduced integrated modular curriculum replacing the annual examination system to semester system.^5^,^6^

The former PM&DC President announced at a conference (AEME) in 2015 that integrated curriculum is a must and all medical and dental colleges should start modifying the curriculum which will be semester system based with integrated assessment. This was followed by new regulations passed by former PM&DC in September 2018, which developed standards of medical education for all medical and dental colleges. At this time they introduced the system for accreditation of medical colleges and medical universities with modifications in teaching hours prescribed for each subject in basic biological and clinical sciences with instructions to revert back to the annual system of examination with immediate effect. This was done due to confusion between general university courses offered through semesters which can be taken at varied times. Medical education is always in continuity and conceptually the course are built upon one another. Henceforth the course cannot be taken at varied times as one cannot move on to an advanced or complex level without having sound knowledge of the previous.

Undergraduate & graduate medical and dental education in Pakistan faces another unique problem of being governed by more than one regulatory body. Former PM&DC was responsible for all regulatory functions, at undergraduate and postgraduate levels, while PMC has shifted some responsibilities to Higher Education Commission (HEC) and some to the College of Physicians & Surgeons, Pakistan (CPSP) thus taking up a limited role. This has resulted in conflicting standards making it difficult for the colleges to comply.

The number of medical and dental colleges in Pakistan has increased tremendously from 22 in 1990^7^ to 177 in 2021.^8^ The accrediting bodies are already struggling with the fact that after 2024 the medical graduates of countries whose accrediting bodies are not recognized by the World Federation of Medical Education (WFME) will not be able to write the United States Medical Licensing Examinations (USMLE) and their names will be removed from the World Directory of Medical Schools. This rapid increase in the number of medical colleges requires trained evaluators and standardization of accreditation/inspection criteria. However, since the criteria are not in the public domain there is a perception is that these decisions are influenced by the political forces.^9^

### Definitions of Politics

There are a multitude of definitions and explanations of the word “politics”. In many places it is described as the art or science of government concerned with guiding or influencing policies or the actions and practices of winning and holding control over a government. In other words, it is the profession, affairs, or businesses concerned with competition between groups or individuals for power and leadership (as in a government). Generally, it is the total complex of relations between people living in a society and their conduct in a particular area of experience especially as seen or dealt with a political point of view for example in office politics, ethnic politics etc.

### Politics may thus be broadly categorized under the following three headings:^4^

1. The academic study of government and the state.
2. The principles inherent in a sphere or activity, especially when it is concerned with power and status.
3. Activities aimed at improving someone’s status or increasing power within an organization.

### Players in the game of politics

English is a flexible language, and it is not uncommon for a word to have multiple related meanings that run the connotative gamut from good to bad. Some of these have been around for a surprisingly long time. Similarly, ‘*Politics’* is a multifaceted word and has a set of fairly specific meanings that are descriptive and nonjudgmental (such as “the art or science of government” and “political principles”), but it can and often does, carry a negative meaning closely related to these (“political activities characterized by artful and often dishonest practices”). The negative sense of *politics*, as seen in the phrase *play politics*,^10^ for example, has been in use since at least 1853, when abolitionist Wendell Phillips declared: “We do not *play politics*; anti-slavery is no half-jest with us.”^11^

The fields of Medicine, dentistry and medical education have attracted the attention of “politicians” since the beginning. Since these topics are important for the health of people, they hold a high value for stakeholders. Hence, political leaders in Pakistan take up an influential role in educational decision making and exert pressure through the regulatory bodies. The political power is exercised through selection of members who in turn influence the policies of admissions, accreditation etc. in PMC^12^. This would be a very positive influence if politics would support and promote good practices to regulate medical and dental education.

### Regulation and accreditation

Medicine and dentistry today are organized through structures that grant the profession considerable autonomy under state protection, while claiming to protect the public from malpractice and quackery.^13^ Accrediting and regulating bodies in most countries ensure that the standards for medical education are in place for the protection of general public. One of the ways of ensuring the implementation of standards is to make transparent the processes and regulations that lead to the establishment and monitoring of the formal curriculum.^14^ The global influences and pressures require adapting and contextualizing pedagogical principles to medical education in Pakistan. For standardization purposes these should be defined at the level of accrediting and regulating bodies. Foremost amongst these core principles is that the curriculum should be contextually relevant and motivating with a combination of knowledge, skills and attitudes to be achieved by the learners, ensuring the development of professional identity in students and making them lifelong learners.^15^ The content should be based on relevant social, cultural and biological constructs and address priority health issues of the country with special emphasis on the issues of marginalized populations.^16^ It is important that the curriculum is built on relevant learning theories and the instruction is based on contemporary learning strategies and the resources available. The assessment practices also need to be contemporary, reliable, valid, feasible and contextual. The curriculum should influence learners to become researchers’ and determine the way research is conducted and communicated.^17^ As pointed out earlier there is no proof that in Pakistan the directions set by regulatory bodies are based on best evidence, needs assessment or determined with involvement of all the stakeholders. Rather these decisions are steered by powerful professionals at the helm of affairs. These include senior medical and dental professionals who are also members of accrediting and licensing bodies and the members of the parliament. These informal groups with members from different disciplines have shared interests, which may not necessarily be educational. The powerful decision makers that are part of these informal groups with allies across nation develop into ‘guilds’. In Pakistan, educational politics has generally demonstrated itself by developing guilds.

The role of accrediting and regulating bodies for medical education is not only in ensuring standards of medical education but also ensuring that medical/dental colleges admit the most appropriate students who would be successful professionals. To safeguard public interest, PMC (formerly PM&DC) has a mission to establish uniform minimum standard of basic & higher qualifications in Medicine & Dentistry throughout Pakistan. For uniformity in all medical and dental colleges across Pakistan, PMC has prescribed that there should be:^18^

• Uniform minimum standard of courses for graduate/postgraduate medical/ dental education.
• Minimum requirements for the content, duration of the courses of study and assessment.
• Uniform conditions for admission to medical/dental courses.
• Uniform minimum qualification and experience for the appointment of teachers/examiners.

The PMC guidelines and policies have given rise to three major concerns. These include admissions, faculty registration and the future of medical graduates wanting to pursue postgraduate medical education in the developed world.

The admission policy revised and implemented by PMC resulted in an uproar by the stakeholders. Under ideal conditions, the decisions for admissions, accreditation and licensure should be guided by the pedagogical principles, but in reality these policies have deep roots in the socio cultural, political and economic environment of the country. However, stakeholders in Pakistan have raised concerns regarding the pedagogical approach being followed in the revised admission policy. A major concern being that it is influenced by the politics of admission.^19^ Sigrid et al., in their editorial discussed that the politics of medical education is not just limited to curricula but also comes in play in the selection of candidates for medical schools.^20^ The conflicting policies at the Federal and Provincial level have led to further confusion and as a result this is the first time in the history of Pakistan that there have been vacant seats in medical and dental colleges.

The issue of faculty registration is also fraught with difficulties as the PMC has relegated the registration of basic science faculty to HEC. The issue is that PMC and HEC have different criteria for appointment at faculty positions. HEC requires a PhD qualification for appointment at the Assistant Professor level, which is a problem as there are very few PhD level programs being offered in the country in these disciplines. The most probable reason being that in order to start a PhD program, one needs three full time faculty members with PhD in the concerned discipline. This raises a serious dilemma of what comes first, chicken or egg. Obtaining a PhD qualification from overseas is exuberantly expensive and not possible for many. Majority of those fortunate to have the resources to study abroad do not return, hence the lacking remains. It is high time that both HEC and PMC need to sit down together and resolve the issues rather than turning a blind eye.

The issue of Pakistani medical graduates aspiring for PGME abroad is also fraught with difficulties. To achieve their goal of standardization of medical education, the World Federation of Medical Education (WFME) has defined standards for undergraduate and postgraduate medical education to provide guidance for enhancing the quality of medical education globally.^21^,^22^ Karle in his systematic review discussed the need for standardization and the issue of who gets what, when, and how.^23^ The need for recognition of accrediting bodies was identified and WFME and the Foundation for Advancement of International Medical Education and Research (FAIMER) decided that the ‘World Directory of Medical schools’ will be revised. Consequently, the Educational Commission for Foreign Medical Graduates (ECFMG) announced that if a Medical Council/Accrediting body, which accredits medical schools in a country is not recognized by the WFME by 2024 then their graduates will not be eligible for the USMLE and hence cannot be employed as physicians in the United States of America (USA).^19^ Hence, in order for our medical and dental graduates to have the avenues of postgraduate education in the developed countries available, the PMC must gain recognition by the World Federation of Medical Education (WFME) at the earliest.

The ruling on April 13, 2022 by the Islamabad High Court of dissolution of PMC and rumors of revival of PM&DC establishes our perspective that such decisions are taken without much thought on their ramifications.^24^ The medical and dental regulatory bodies in Pakistan have still not complied with the accreditation requirements established by the ECFMG in 2010 with an earlier deadline of 2023, which has recently been moved to 2024. The politics keeps us oscillating between PMC, PM&DC & HEC without realizing that time is running out and that our students will not be able to enter international postgraduate medical education programs if correct decisions are not taken now. It is high time that the decision makers should aim ***to connect the pedagogy of education and the context of Pakistan*.**

### What is Pedagogy?

Pedagogy is the art and science of learning and in all teaching and learning situations, be it children or adults, the relationship between teachers and students is referred to as pedagogy.^25^,^26^ The core principles of Pedagogy are embedded in the theoretical perspectives of learning and can be contextualized by any institution ensuring the use of contemporary instructional strategies for enhanced learning. Mann KV, stated in her paper that pedagogy in medical education has been shaped by various theories that include theories with a behaviorist, humanists, cognitivist, social and constructivists’ underpinnings^27^-^29^.

To understand the process of decision making at PMC in addition to pedagogy and the politics of education we have to consider the informal formation of guilds.

### What are Guilds?

Guilds are Monopolistic^30^ aiming to exert exclusive possession or control of the concerned trade in a commodity or service. They are not necessarily negative as their paternalistic approach (protecting their own)^31^ can be productive in raising the standards of quality of the occupations or service.^32^ However, the guilds may be oblivious to their effects and need.^33^

The “truly autonomous” occupation in health services is medicine, and that its autonomy is sustained by the dominance of its expertise in the health division of labour. Ford, pointed out that these effects of medical dominance ‘ can be the cause of bad experiences by patients.^34^ To combat this medicine today is organized through structures that grant the profession considerable autonomy under state protection while claiming to protect the public from malpractice and quackery.^13^ These structures are in the form of medical associations, accrediting, regulatory and licensing bodies.

Medical Associations try to control the practice and with extensive networking the control may go further than just appreciating or supporting. If we look at the case of specialist’s associations, they prefer their own trainees to join the club, which may sometimes go beyond support of merit and become biased.^3^

### Pakistani Guilds

Many medical guilds are working in Pakistan including those formed to regulate the professions such as the PMC or HEC and those concerned with training postgraduates such as CPSP. They are engaged with setting regulations, such as defining admission criteria, curriculum, competencies and setting boundaries with respect to who can do what kind of practice thus claiming to ensure the quality of care and patient safety.

Within these organizations (PMC/HEC/CPSP) guilds are formed by the professionals in positions of power. We need to recognize that although this formation may not be totally erroneous, these guilds need to reflect on their supportive and controlling practices as guardians of the profession. So far, these guilds are neither recognized nor acknowledged either within the respective organizations or outside in Pakistan.

### Putting it all together

This discussion highlights the role of politics, pedagogy, and guilds in dealing with the burning issues of medical education in Pakistan. Alliances are formed between professional leadership at regulatory licensing and accrediting bodies with the relevant political leadership. The guilds at the level of these bodies take over and following the principles of guilds, make changes in accreditation and regulations. Decision made through these alliances have implications that are not thought through and not based on principles of pedagogy. In Pakistan, the PMC is striving to transform the educational system however, unfortunately during the process of transformation, it has blatantly ignored the principles of pedagogy as the basis for setting up educational regulations. The development of policies and decisions lack consultation with relevant stakeholders and hence fail to take into account the feasibility and practicality of the decisions. These decisions have long term effects on the future medical and dental graduates and eventually on the overall health of the population.

There is a need for a radical change at all levels. At the level of PMC, they include professionalization of the profession, institutionalization of standards and developing competency framework for medical and dental graduates. However, the need to straight jacket and micromanage educational programs stifles creativity and innovation. A combination of this and with a lack of stakeholders’ involvement and consideration of ground realities is a recipe for failure. Therefore, the institutions should be given freedom to design and develop innovative learning strategies as needed based on sound pedagogical principles. Moreover, the accrediting & licensing bodies should focus on reinforcing the standards and ensure that the competency framework is reflected in the curriculum and assessed for licensure.

## CONCLUSIONS

The interplay of market forces, politics, medical advocacy and pedagogy should complement each other for better quality of medical education and community health. The market forces, politics of medical education vis-a-vis the guilds should not take over the pedagogy of medical education. Medical Advocacy will always be required for quality assurances and making appropriate changes in admissions, faculty regulation, licensing examinations, curriculum modifications and accreditation, “The Medical Educationists” in Pakistan should ensure that medical advocacy should take into account the needs of the community and betterment of medical education. Additionally, the medical educationist and influential members at regulatory bodies should learn from previous guilds and refrain from strengthening this phenomenon.

### Author’s Contribution:

**LAB:** Concept, first draft, final revision.

**SKA:** Critical review, revision, response to reviewer’s comments.

**SS:** Literature review, writing introduction, review of references.
